# What Study Abroad Holds out for the Graduate

**Published:** 1907-09-07

**Authors:** 


### WHAT STUDY ABROAD HOLDS OUT FOR THE GRADUATE.

### Continental Schools.

The benefits which the graduate may gain by spending a few months or weeks at some Continental centre are many and important. The change of climate, scenery, and environment generally will react beneficially upon him; the study of the language, manners, and customs of his new hosts will be a matter of daily interest to him. At the schools he will meet with new methods of treatment; he will find prejudices existing against certain methods which he thought were as firmly fixed on generally accepted principles as the theory of the circulation of the blood or of the revolution of the earth round the sun; and he will find also methods in use which in his own school his teachers had taught him were absurd and valueless. If he is unsympathetic, insular, and aggressive he will reject at once what he hears affirmed. To him Sayre's bandage will remain for ever superior to Hennequin's, which the French hospitals use, and strychnine eternally the superior of camphor in heart failure. But it is not to the unsympathetic, aggressive, insular graduate that we recommend foreign study, for he will gain little from it save material enough to strengthen and confirm him in his dislikes. To the man who can discriminate, who can nicely weigh the pros and cons of two methods of treatment, neither accepting the one because it is home-grown and endemic, and therefore superior to everything else, nor rejecting the other because it is foreign, outlandish, and therefore bound to be wrong; who can, above all, judge sympathetically, going by results not by alleged reasons, such a course of study cannot but prove of the greatest possible value.

### Preliminary Considerations.

Before venturing to attach himself to a foreign school the graduate will have to consider very carefully the question of language, residence, time, and expense. Usually only a three months' course at the most can be indulged in, and most students will have to be satisfied with a much shorter course. A great deal, however, may be accomplished even in that limited period. Many a man does excellent work abroad during his annual holiday, and a visit to a hospital or a morning's attendance at a foreign clinic will, in default of other opportunities, prove useful to the graduate. The question of expense is not a very important one. In all the great medical centres of the Continent living, for the student at least, is inexpensive. Cheap lodgings and board can be had at prices ranging from 12s. 6d. to 35s. per week, and for short visits the pensions and smaller hotels do admirably. The fees payable for attendance at classes and demonstrations are generally very much lower than those paid for corresponding classes in England, and extras, on the whole, do not total up to much. Where two or more friends share expenses the cost of a foreign course may be very much reduced. The language question is one which the graduate should consider carefully. In most of the large European hospitals English classes are held, during the vacations, for foreign graduates, mainly English and American. The professors usually know and talk English, and the demonstrators make a point of learning the language. But the graduate would be well advised to drop for the time being his home language along with his old text-books, and whatever old prejudices he may have gathered together during his student days, and to apply himself seriously and whole-heartedly to mastering the language of the out-patients at the place where he is studying. German and French and Italian are not very difficult languages to acquire, and a knowledge of one of the three will be of invaluable assistance both while abroad and afterwards.

*France.*—In France the opportunities for English Most of the hospitals are open to foreign graduates on payment of special fees. For an ordinary visit or attendance at a demonstration the production of the visitor's card is usually quite sufficient. The following hospitals have special lectures and classes, particulars of which may be obtained on application to the respective Deans:—Paris: Hôtel-Dieu, 560 beds; La Pitié, Charité, Necker, Lariboisière, Beaujon, Laennec, Andral, all general hospitals varying from 300 to 800 beds; Saint-Louis, Midi, Lourcine, Clinique, Maison de Santé, Trousseau, Pasteur, special hospitals with from 100 to 250 beds. The clinical staff attached to these are members of the Faculty of Medicine of the University of Paris, and full notice of lectures and demonstrations are usually posted on the hospital walls. At French hospitals out-patients are seen from 7.30 to 9 in the morning; operations are usually in the morning, and demonstrations and lectures soon after noon. In Paris special classes are held in forensic medicine and medical jurisprudence, and the introductory lectures to the various courses in surgery, medicine, and midwifery are usually exceptionally instructive. Excellent courses are given at some of the provincial centres in France. Caen and Lille, both university towns, attract many English students. Orleans and Marseilles possess good hospitals where foreign graduates are allowed to study.

*Germany.*—All the German Universities extend their cordial hospitality to graduates of sister universities and to diplomates of learned societies. In Berlin, Erlangen, Halle, Tubingen, Wurzburg, Bonn, and, above all, in Heidelberg, the student will find himself soon admitted to the full into the privileges and rights of the ordinary abiturient. The professors are invariably extremely courteous: they go out of their way to help and assist the stranger, and they put willingly at his disposal, for purposes of study, their wards and their lectures. At most of the better-known centres special classes are held for foreign students: these classes are essentially "post-graduate" classes; they are practical and sometimes include laboratory work. For pure medicine the graduate will probably find himself best catered for at Munich and Berlin: in surgery Halle and Heidelberg offer excellent opportunities to the student for becoming familiar with German methods of work. For research work Germany offers unrivalled opportunities.

*Austria.*—Vienna, Prague, and Innsbruck cater for the American and English graduate by advertising special classes in otology, ophthalmology, laryngology, and allied subjects during the vacation. These vacation courses are held at the various special clinics, by some well-known expert who usually lectures and demonstrates in English and allows students to do practical work under him. The fees for such vacation courses are a trifle higher than those charged for ordinary courses. Living in Vienna is somewhat more expensive than at other centres, and the English student community there is usually large, Americans being specially well represented.

*Italy.*—No special facilities are put in the way of the foreign student, but he is always welcome at every university and in every hospital. At Milan the unrivalled Ospedale Maggiori well repays a few weeks' study. At Rome, Turin, Florence, and Venice there are excellent hospitals and periodical courses of lectures on certain subjects, which may be attended on production of evidence of registration or on payment of a fee. As in Paris, a speciality is made of medico-legal study, and as has already been said the clinical teaching and pathological demonstrations are excellent. At the large new hospital at Rome special post-graduate classes are now being held.

*Switzerland.*—Centres to be recommended are Basle, Zurich, and Berne, at each of which vacation courses are held.

*Holland.*—No facilities are extended to the graduate, but, as in Italy, he is everywhere courteously made welcome. The best clinical teaching is to be found in Amsterdam, Leyden, and Groningen.

*Russia.*—As a field for post-graduate study Russia has possibilities, but they are not sufficiently well-known or attractive to appeal to the average graduate. At St. Petersburg alone does the foreign student obtain special facilities for such study, but there he enjoys unrivalled opportunities for studying special subjects. The teaching in surgery, midwifery, and orthopædics is specially good in Russian centres.

